# BAFF is involved in macrophage-induced bortezomib resistance in myeloma

**DOI:** 10.1038/cddis.2017.533

**Published:** 2017-11-02

**Authors:** Jing Chen, Donghua He, Qingxiao Chen, Xing Guo, Li Yang, Xuanru Lin, Yi Li, Wenjun Wu, Yang Yang, Jingsong He, Enfan Zhang, Qing Yi, Zhen Cai

**Affiliations:** 1 Bone Marrow Transplantation Center, The First Affiliated Hospital, School of Medicine, Zhejiang University, Hangzhou, Zhejiang, China; 2 Department of Cancer Biology, Lerner Research Institute, Cleveland Clinic, Cleveland, OH, USA

## Abstract

We aimed to characterize the role of B-cell activating factor (BAFF) in macrophage-mediated resistance of multiple myeloma (MM) cells to bortezomib (bort), and to further understand the molecular mechanisms involved in the process. First, we detected BAFF and its three receptors on myeloma cells and macrophages using the quantitative reverse transcriptase-polymerase chain reaction and flow cytometry. The secretion of BAFF was tested in patients with MM, MM cell lines, and macrophages. The ability of macrophages to protect MM cells from bort-induced apoptosis was significantly attenuated using BAFF-neutralizing antibody in the co-culture system or knocking down the expression of BAFF in macrophages with small interfering RNA. We also showed that the MM–macrophage interaction through BAFF and its receptors was primarily mediated by the activation of Src, Erk1/2, Akt, and nuclear factor kappa B signaling and the suppression of caspase activation induced by bort. Our data demonstrated that BAFF played a functional role in the macrophage-mediated resistance of MM cells to bort, suggesting that targeting BAFF may provide a basis for the molecular- and immune-targeted therapeutic approach.

Multiple myeloma (MM) is a universally clonal B-cell neoplasm characterized by the expansion of malignant plasma cells in the hematopoietic bone marrow (BM).^1^ MM cells are protected from both spontaneous and drug-induced apoptosis as a consequence of adhesion to certain microenvironmental components.^2^ Bortezomib (bort, Velcade) is one of the best effective treatments for MM. It has simultaneously targeted MM cells and their closely supportive BM environment.^3^ Although initial advantages of bort treatment of MM including higher overall response rates are promising, a number of patients develop a resistance to it over time.^4, 5^

To date, the mechanism of bort resistance is unknown. Recent studies have shown that MM cells do manifest a clonal heterogeneity,^6^ and their mutation or overexpression of bort-binding protein at the *β*5 proteasome subunit^7^ may result in the acquired resistance to bort. The upregulation of insulin-like growth factor-1, heat shock proteins, *β*-catenin/Wnt, and c-Met/phosphor c-Met has been suggested.^8^ Bort resistance has also been related to the activation of prosurvival autophage^8^ and alterations in bone marrow stromal cells (BMSCs).^9^

Tumor-associated macrophages (MΦs) are the prominent components in the stroma. They provide a favorable microenvironment for tumor cells by cross-talking with other stromal cells and thus promote tumor growth, progression, and metastasis. In MM, MΦs could induce drug resistance by protecting tumor cells from chemotherapy-induced apoptosis, and microarray analysis has ranked the top 250 paired genes including B-cell activating factor (BAFF) that may play a role in the MΦs–MM cell interaction.^10^

Investigators have reported that myeloma cells express transmembrane activator and calcium-modulator and cyclophilin ligand interactor (TACI) and B-cell maturation antigen (BCMA), two genes coding for receptors of BAFF (also known as Blys).^11^ BAFF, a member of the tumor necrosis factor (TNF) family, was identified as a key factor in the normal B-cell biology. It also enhances the survival of various B lymphocyte malignancies, including MM.^12, 13, 14^ It can act as a membrane-bound or proteolytically cleaved soluble form displaying typical features of type II transmembrane protein.^15^It is expressed predominantly by stromal compartment including osteoclast, MΦs, dendritic cells, and some T cells.^16^ Some studies have found that the tumor microenvironment secretes BAFF.^17^

Therefore, we choose to investigate the significance of BAFF in MΦ-mediated MM bort resistance. We showed that primary myeloma cells and MM cell lines expressed BCMA and TACI heterogeneously. The expression of BAFF in MΦs increased compared with monocytes. The BAFF-neutralizing antibody or knockdown of BAFF further attenuated the MΦ-induced bort resistance of MM cells. Thus, we try here to define a key role of BAFF, which is essential for MΦ-mediated bort resistance of MM cells.

## Results

### Expression of BAFF and its receptors detected in MM cells

**Figure 1:**
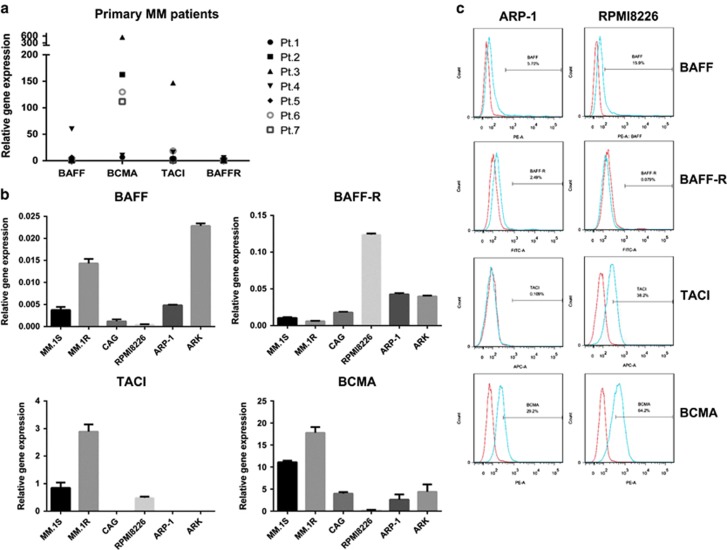
Expression of BAFF and BAFF-Rs in myeloma cells. The mRNA expression of BAFF and its receptors (BAFF-R, TACI, and BCMA) were detected using RT-PCR in seven CD138+ purified primary MM cell samples (**a**) and in six MM cell lines MM.1S, MM.1R, CAG, RPMI8226, ARP-1, and ARK (**b**). The cell surface expression of BAFF and its receptors in ARP-1 and RPMI8226 were detected by flow cytometry using anti-BAFF, anti-BAFF-R, anti-TACI, and anti-BCMA (blue lines) for 15 min at room temperature. Red lines indicate isotype Ig controls, done for each sample (**c**)

Our study first detected the expression of messenger RNA of BAFF and its three receptors, BCMA, TACI, and BAFF-R, from seven patients with MM and in six MM cell lines using qRT-PCR to evaluate the relevance of BAFF signaling in MM drug resistance. As shown in Figure 1a, tested primary MM cells express BAFF heterogeneously. Among the receptors of BAFF, the expression of BCMA was significantly higher than that of TACI, with BAFF-R being the lowest: the maximum arbitrary units of BAFF, BCMA, TACI, and BAFF-R were 50, 600, 150, and 5, respectively. Similar results were observed in the six MM cell lines MM.1S, MM.1R, CAG, RPMI8226, ARP-1, and ARK (Figure 1b). Overall, the heterogeneous expression of BAFF and its receptors was consistent with that reported by Yu-Tzu *et al.*^18^ Then, the surface expression of BAFF and its receptors in the two MM cell lines ARP-1 and RPMI8226 was examined using flow cytometry. Notably, ARP-1 and RPMI8226 expressed higher levels of BCMA and TACI, lower levels of BAFF, and virtually undetectable BAFF-R, suggesting that altered expression of BAFF and its receptors might contribute to the process of MM cells resistant to apoptosis.

### PBMC-induced MΦs and expression of BAFF and its receptors in MΦs

**Figure 2:**
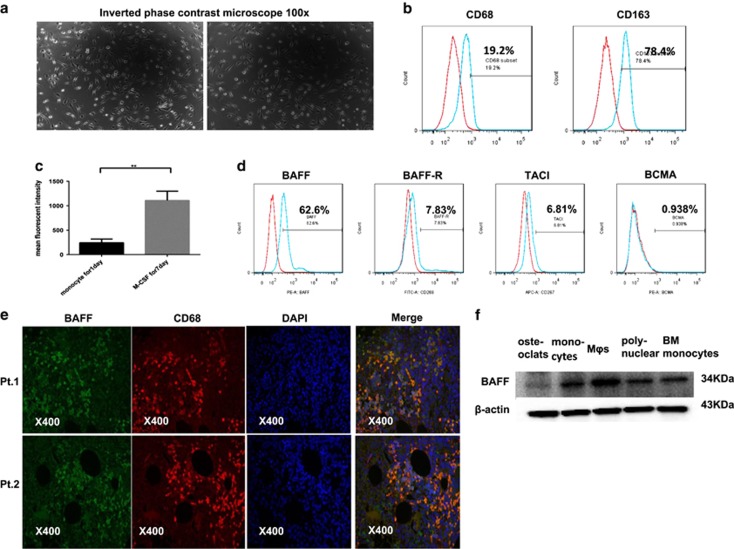
PBMC-induced MΦs and the expression of BAFF and BAFF-Rs in MΦs. Monocytes from healthy donors were cultured in RPMI-1640 supplemented with 10% FBS in the presence of M-CSF (10 ng/ml) for 7 days. (**a**) M2-type MΦs adhered to the six-well plates and had a spindle-like morphology. (**b**) MΦs were positive for CD68 and CD163 by flow cytometry analysis. (**c**) Monocytes and monocyte-induced MΦs from seven blood donors were detected for the expression of BAFF by the flow cytometry analysis. Numbers represent the mean fluorescent intensity. (**d**) Cell surface expression of BAFF and its receptors in MΦs were detected by flow cytometry. (**e**) Expression of BAFF on primary MΦs from BM aspirates of patients with MM (*n*=2) were detected using immunofluorescence. (**f**) Expression of BAFF on monocyte-derived osteoclast, monocytes, MΦs, polynuclear cells, bone marrow monocytes were detected by Western blot

Macrophage colony-stimulating factor (M-CSF) is a key homeostatic growth factor involved in the maintenance and differentiation of MΦs.^19^ It is well established that M-CSF preferentially stimulated M2-like MΦ phenotypes.^20, 21, 22, 23^ M2-like MΦs had characteristics of spindle-like cells and relatively high expression of CD163 surface marker.^24^ In our study, MΦs were harvested from peripheral blood monocytes (PBMCs) of healthy donors, which were incubated for 7 days with M-CSF. We observed that the cultured MΦs were adherent to the six-well plates and had a spindle-like morphology (Figure 2a). The expression of CD68 and CD163 of MΦs from different donors was (24.6±0.39%) and (78.4±0.67%), respectively. The flow cytometry analysis of the two CD molecules in MΦs is shown in Figure 2b. Simultaneously, the expression of BAFF was measured during MΦ differentiation. As shown in Figure 2c, MΦ had increased expression of BAFF compared with monocytes (*P*<0.05), and in monocyte-derived MΦ, BAFF had relatively high expression whereas its receptors were barely detected by flow cytometry (Figure 2d). We also identified the expression of BAFF in CD68+ monocytes/MΦs from BM aspirates of patients with MM using immunofluorescence (Figure 2e). Moreaux *et al.*^17^ had pointed out BAFF were mainly expressed by osteoclasts and BMSCs in the bone microenvironment. Concerning the induction of MΦs in our experiment, we simultaneously detected BAFF expression in monocyte-derived osteoclast, monocytes, MΦs, polynuclear cells, BM monocytes by western blot. Of major interest, MΦs largely expressed BAFF while osteoclasts weekly expressed BAFF (Figure 2f).

### BAFF production by BM cells, MM cell lines, and MΦs

**Figure 3:**
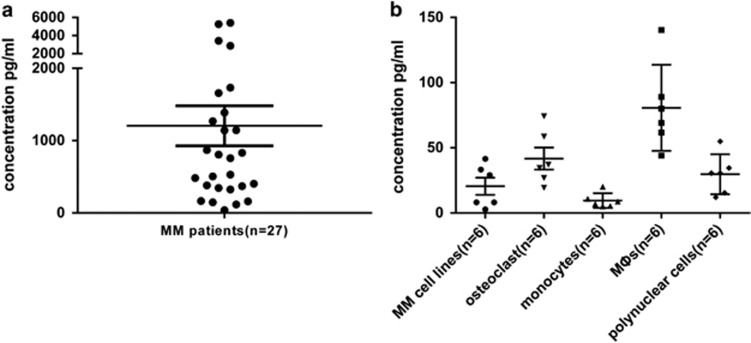
Expression of soluble BAFF. The supernatants of (**a**) BM of 27 patients with MM and (**b**) MM cell lines, osteoclasts, monocytes, MΦs and polynuclear cells from six donors were collected for detecting BAFF using ELISA

As BAFF is a secreted protein, we explored the presence of BAFF protein in the supernatants of BM of patients with MM, MM cell lines, and osteoclasts, monocytes, MΦs, polynuclear cells from various donors. Using ELISA, median soluble BAFF levels were 1306.9, 20.5, 41.7, 9.60, 80.7, and 29.7 pg/ml in culture supernatants of BM of patients with MM, MM cell lines, osteoclasts, monocytes, MΦs and polynuclear cells, respectively, showing the presence of the soluble form of BAFF (Figures 3a and b).

### PBMC-induced MΦs were insensitive to bort and protected MM cells from bort-induced apoptosis

**Figure 4:**
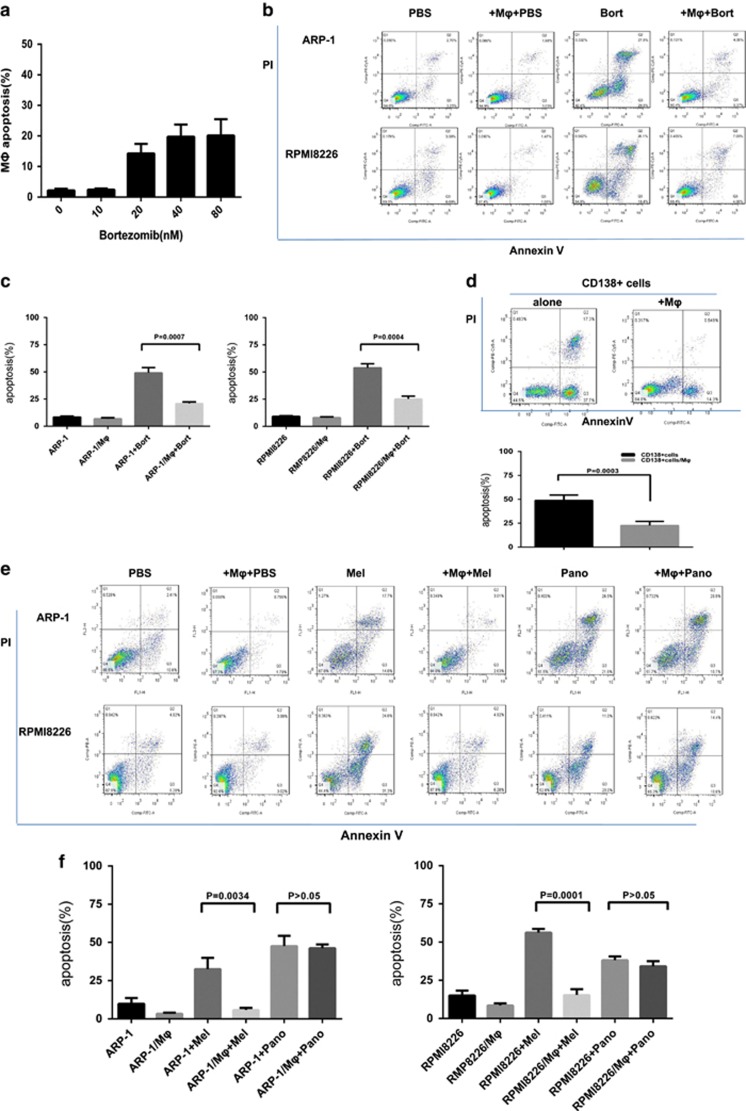
Effect of bort on MΦs and MM cells. (**a**) Effect of bort on MΦs. Bort has small proapoptotic effect in MΦs. Values are presented as means±S.D. (**b**,**c**) ARP-1 and RPMI8226 cells were cultured alone or co-cultured with MΦs in the absence or presence of bort (5 and 10 nM for ARP-1 cells and RPMI8226, respectively; 24 h). Apoptosis was evaluated by flow cytometry with Annexin V/PI staining. (**b**) A representative result showing that MΦs reduced the bort-induced apoptosis of ARP-1 and RPMI8226 cells. (**c**) The protective effect of MΦs from different donors is analyzed as means±S.D. (**d**) A representative and summarized result showing that MΦs prevented spontaneous apoptosis of primary CD138+ plasma cells. Values are presented as means±S.D. (**e**,**f**) ARP-1 and RPMI8226 cells were cultured alone or co-cultured with MΦs in the absence or presence of Mel (15 and 20 *μ*M for ARP-1 cells and RPMI8226, respectively; 24 h) or Pano (250 and 300 nM for ARP-1 cells and RPMI8226, respectively; 24 h). Apoptosis was evaluated by flow cytometry with Annexin V/PI staining. (**e**) A representative result showing that MΦs reduced apoptosis of ARP-1 and RPMI8226 cells from melphalan rather than panobinostat. (**f**) The protective effect of MΦs from different donors is analyzed as means±S.D.

Because BM microenvironment contributes to the drug resistance of plasma cells,^25^ we wondered whether MΦs mediated the resistance of MM cells to bort. The role of PBMC-derived MΦs *in vitro* was determined via bort-induced apoptosis of ARP-1, RPMI8226, and CD138+ plasma cells from patients with MM. We first investigated the direct function of bort on MΦs. Bort (range 0–80 nM) had small effect in inducing apoptosis of MΦs (Figure 4a). Besides, MΦs co-cultured with ARP-1 (bort, 5 nM) and RPMI8226 (bort, 10 nM) significantly weakened bort-induced apoptosis (Figures 4b and c). Bort concentration was determined according to the inhibitory concentration 50% of ARP-1 and RPMI8226 (data not shown). Moreover, CD138+ plasma cells from four patients with MM, which were susceptible to spontaneous apoptosis *in vitro*, were obviously protected by MΦs (Figure 4d) when co-cultured with MΦs, suggesting the protective effect of MΦs. We also extended our study to the conventional agent melphalan (Mel) and histone deacetylase inhibitors (HDACi) panobinostat (Pano). The results showed that MΦs protected ARP-1 and RPMI8226 from Mel-induced apoptosis under the co-culture condition. However, the protective effect was no longer observed when tested MM cells were treated with Pano (Figures 4e and f).

### BAFF was indispensable for MΦ-mediated bort resistance of MM cells

**Figure 5:**
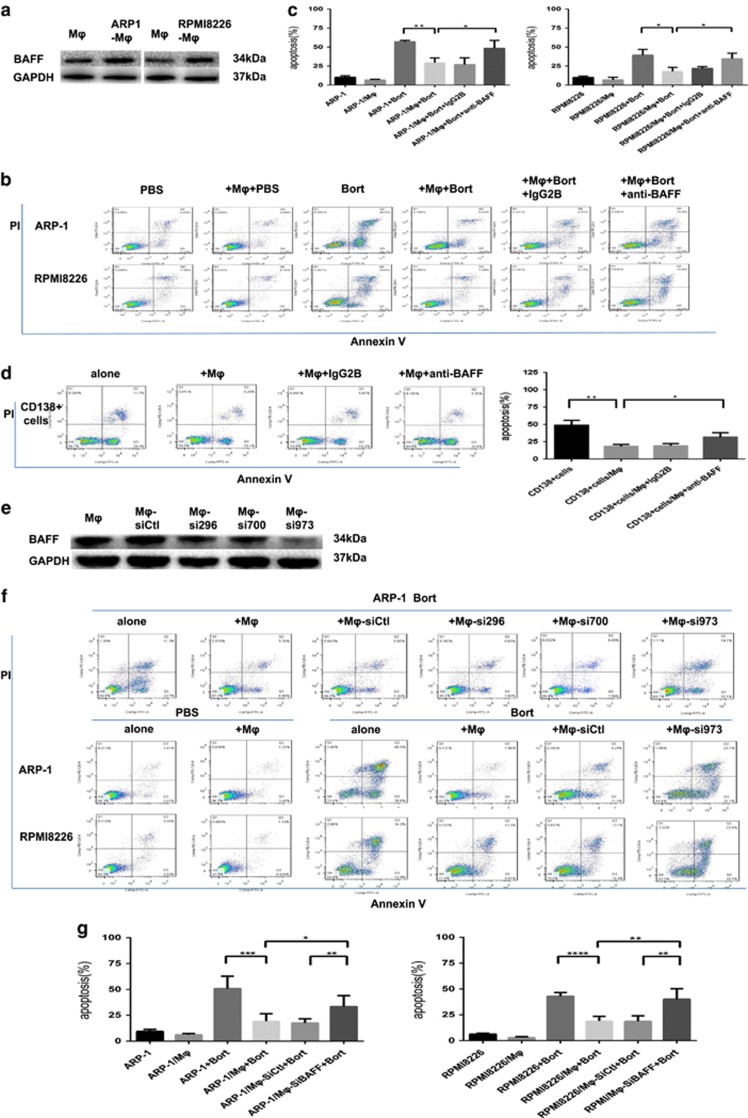
BAFF is indispensable for MΦ-mediated MM bort resistance. (**a**) Lysates from MΦs either cultured alone or co-cultured with MM cell lines (ARP-1 and RPMI8226) were detected for the expression of BAFF protein using an anti-BAFF antibody by Western blot, with GAPDH used as a loading control. Apoptosis was evaluated by flow cytometry with Annexin V-FITC/ propidium iodide staining. (**b**) A representative result showing the percentage of bort-induced apoptotic MM cells (5 nM ARP-1 and 10 nM RPMI8226) in direct co-culture with MΦs, in the presence of BAFF-neutralizing antibody (20 *μ*g/ml) or control IgG2B (20 *μ*g/ml). (**c**) Reduced protective effect of MΦs in the presence of BAFF-neutralizing antibody is analyzed as means±S.D. (**d**) Results showing BAFF-neutralizing antibody attenuated the effect of MΦs in protecting primary CD138+ plasma cells from spontaneous apoptosis. Values are presented as means±S.D. (**e**) MΦs treated with BAFF-specific siRNAs showed a diverse reduction of BAFF protein compared with nontargeting siRNA (control) at 72 h using Western blot, with GAPDH as a loading control. (**f**) A representative result showing bort-induced apoptosis on ARP-1 cells co-cultured with MΦs following the BAFF-specific·siRNAs using flow cytometry analysis. The BAFF knockdown effect resulted in reduced ability of MΦs in protecting MM cells. (**g**) Result showing percentage of bort-induced apoptotic MM cells (ARP-1 and RPMI8226) in direct co-culture with BAFF-knocked down MΦs. Values are presented as means±S.D.*P<0.05, **P<0.01, ***P<0.001, ****p<0.0001

A previous study provided the gene expression profile data of MM cells and MΦs cultured alone or co-cultured, 250 paired genes were differentially expressed.^10^ Based on these data, we hypothesized that BAFF (on MΦs) and its receptors (on MM cells) played a role in the MΦ-mediated bort resistance of MM cells. MΦs were cultured alone or co-cultured with MM cell lines for 24 h, the suspended MM cells were removed and washed with phosphate-buffered saline (PBS) to obtain pure MΦs, we found MΦs in co-cultured condition led to an increased expression of BAFF as detected by western blot (Figure 5a). We interrupted the interaction between BAFF and its receptors using BAFF-neutralizing antibody, and then examined MΦ-mediated protection. As shown in Figures 5b and c, the BAFF-neutralizing antibody, but not control IgG2B, repressed the MΦ-mediated bort resistance of MM cells (ARP-1 and RPMI8226) and partially restored MM cell sensitivity to bort in direct co-culture with MΦs. Similar findings are shown in Figure 5d in which BAFF-neutralizing antibody attenuated the MΦ-mediated spontaneous apoptosis of CD138+ plasma cells compared with control IgG2B.We also examined MΦ-mediated MM drug resistance in BAFF-knocked down MΦs. MΦs were transduced with siRNAs targeting BAFF (TNSF13B-Homo-296, TNSF13B-Homo-700, and TNSF13B-Homo-973) or control nontargeting siRNA. The transduction of BAFF-specific siRNAs reduced protein levels of BAFF in MΦs differently (Figure 5e). TNSF13B-Homo-973 conferred that significant BAFF knockdown resulted in the reduced ability of MΦs in protecting MM cells (ARP-1 and RPMI8226) from bort-induced apoptosis (Figures 5f and g; compared with sictl-MΦs). Thus, our findings indicated that BAFF (on MΦs) and its receptors (on MM cells) played a profound role in MΦ-mediated bort resistance of MM cells. Similarly, BAFF-knocked down MΦs were not protecting MM cells against Mel-induced apoptosis to a large extent (Supplementary Figures 1A and B).

### Effect of BAFF on signaling pathway in co-cultured MM cells

**Figure 6:**
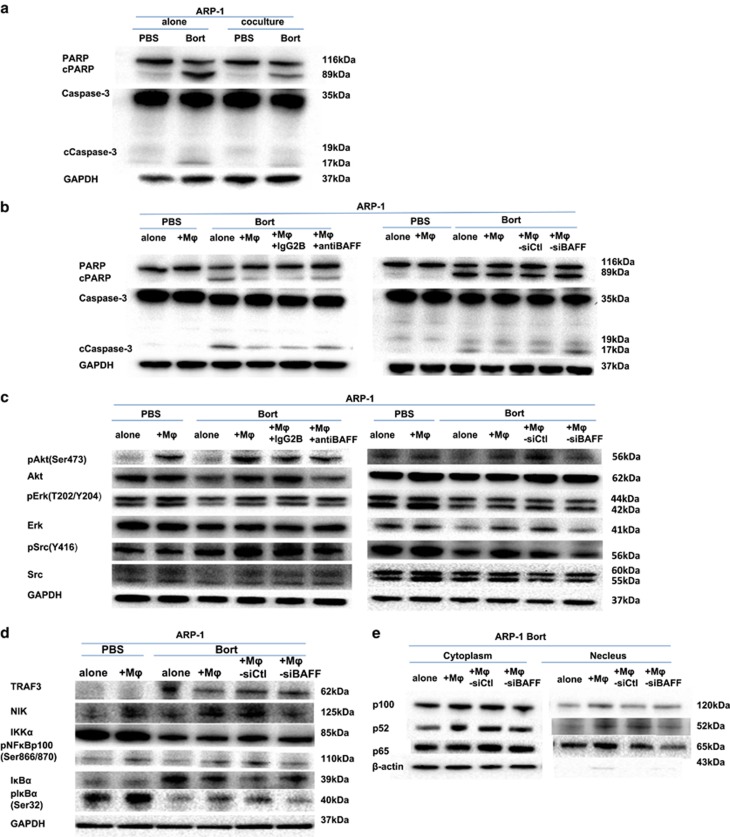
Involvement of BAFF in MΦ-mediated protection of ARP-1 cells. Western blot analyses showed that (**a**) ARP-1 cells cultured alone or co-cultured in direct contact with MΦs were detected for activation and cleavage of PARP and caspase-3, either PBS-treated or bort-treated (5 nM, 24 h). (**b**) ARP-1 cells cultured alone or co-cultured in direct contact with MΦs in the presence of BAFF-neutralizing antibody or control IgG2B and ARP-1 cells cultured alone or co-cultured in direct contact with sictl-MΦs or siBAFF-MΦs were detected for activation and cleavage of PARP and caspase-3, either PBS-treated or bort-treated (5 nM, 24 h). (**c**) Expression of pAkt (Ser473), Akt, pErk1/2 (T202/Y204), Erk1/2, pSrc (Y416), Src, and GAPDH in ARP-1 cells cultured alone or directly co-cultured with MΦs in the presence of BAFF-neutralizing antibody or control IgG2B and in ARP-1 cells cultured alone or directly co-cultured with sictl-MΦs or siBAFF-MΦs, either PBS-treated or bort-treated (5 nM, 24 h). (**d**) Expression of TRAF3, NIK, IKK*α*, I*κ*B*α*, and pIκB*α* (Ser32) in ARP-1 cells directly co-cultured with sictl-MΦs or siBAFF-MΦs, either PBS-treated or bort-treated (5 nM, 24 h). (**e**) Processing of p100 to p52 and translocation of p52 or p65 to the nucleus in ARP-1 cells directly co-cultured with sictl-MΦs or siBAFF-MΦs, bort-treated (5 nM, 24 h)

To learn if the intracellular survival signaling was stimulated by MΦ/MM co-culture that conferred bort resistance of MM cells *in vitro*, first, apoptotic cell characteristics of PARP and caspase-3 cleavage were detected using western blot in MM cells under co-culture conditions either with BAFF-neutralizing antibody or control IgG2B in the presence of bort. The result showed that in ARP-1 cells co-cultured with MΦs, bort-induced PARP and caspase-3 cleavage had high repression (Figure 6a). However, neutralization of BAFF under co-culture conditions or ARP-1 cells co-cultured with BAFF-knocked MΦs (Supplementary Figure 2A) had moderate elevation of these apoptotic proteins (Figure 6b). Next, the possible signaling mediators were examined in MM cells. MΦs were found to activate phosphorylated Akt, Erk1/2 kinase, and Src in ARP-1 cells treated with bort, which had lower levels of p-Akt, p-Erk1/2, and p-Src when treated with BAFF-neutralizing antibody. Similar findings were also observed from BAFF-knocked-down MΦs (Supplementary Figure 2B) co-cultured with ARP-1 cells, suggesting that BAFF played a role in MΦ-mediated bort resistance through Akt, Erk1/2 and Src pathway activation (Figure 6c).

A previous study explained that the BAFF promoter was an essential activation element of nuclear factor kappa B (NF-*κ*B) transcription triggered by the adhesion of MM cells to BMSCs.^26^ NF-*κ*B2 activation relies on both NIK (NF-*κ*B-inducing kinase) and its downstream kinase IKK*α* with the persistent degradation of TRAF3 and increased expression of NIK. It also involves the processing of p100 to p52 and translocation of p52 to the nuclear fraction.^27, 28, 29^ Our present study found that BAFF-knocked-down MΦs (Supplementary Figure 2C) co-cultured with ARP-1 cells partly repressed the activation of NF-*κ*B2 (Figure 6d). We also observed the degradation and phosphorylation of the inhibitor of kappa B*α* (I*κ*B*α*) and p65 translocation to the nucleus, implying the activation of the canonical pathway of NF-*κ*B (Figures 6d and e). The signaling pathway was not activated further by co-culture with BAFF-knocked-down MΦs (Figures 6d and e). These results indicated that BAFF-induced bort resistance of MM cells co-cultured with MΦs was conducted via activation of both classical and alternative NF-*κ*B pathways.

### MΦ-mediated bort resistance of MM cells *in vivo*

**Figure 7:**
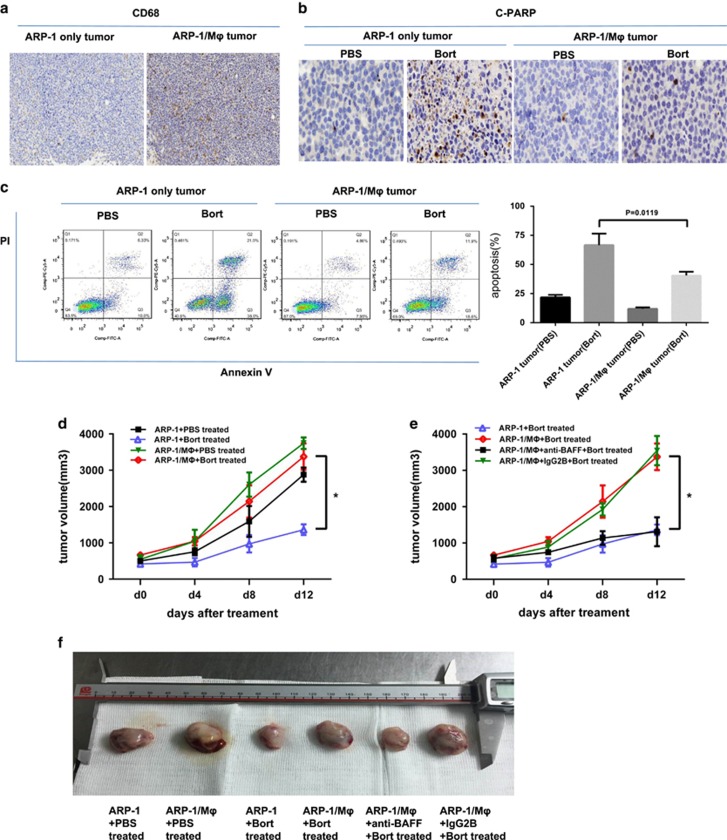
*In vivo* effect of MΦ-mediated MM bort resistance in the myeloma NOD–SCID mouse model. (**a**) Human MΦ infiltration in tumors from ARP-1 cells (ARP-1 only tumor) and ARP-1/monocytes (ARP-1/MΦ tumor) was detected by immunohistochemistry staining of CD68. Tumors from myeloma-bearing NOD–SCID mice treated with bort (2 *μ*g/mouse every 3 days for 2 consecutive weeks) or PBS (served as controls) were detected for apoptotic cells (**b**) using immunohistochemistry staining with anti-cPARP antibody and (**c**) flow cytometry staining with Annexin V-FITC/propidium iodide. (**d**) Tumor burdens under different groups were detected as tumor volume. (**e**) Tumor volume of bort-treated myeloma-bearing NOD–SCID mice (n=5/group) treated with BAFF-neutralizing antibody (100 mg/mouse, every 3 days for 2 consecutive weeks) or control IgG2B. *P<0.05. (**f**) Tumor volume in different treatment groups

The human MM-NOD-SCID mouse model was used to evaluate whether *in vivo* environment corresponded to *in vitro* findings that MΦs could protect myeloma cells from bort-induced apoptosis. ARP-1 cells and ARP-1 mixed with monocytes were subcutaneously injected into the flanks of NOD-SCID mice. We enumerated MΦ infiltration in a tumor by immunohistochemical analysis using the anti-human CD68 antibody (Figure 7a). Mice bearing ARP-1 tumor alone or ARP-1 tumor mixed with human MΦs were treated with bort every 3 days to assess bort-induced cell death *in vivo*. After treatment for 2 consecutive weeks, the mice were killed and the tumors were harvested. Then, the apoptotic cells of tumor masses were detected by immunohistochemistry staining with the anti-cPARP antibody (Figure 7b) and flow cytometry analyses for Annexin V-FITC/propidium iodide(PI)-positive cells (Figure 7c). The tumor generated by ARP-1 alone cells had more positive staining of cPARP and Annexin V/PI. These results supported that MΦs could protect MM cells in the presence of bort *in vivo*. Consistently, this study found that mice bearing ARP-1/MΦ cells had a larger tumor volume in the presence and absence of bort (Figure 7d), indicating that MΦs manifested compromised therapeutic effects of bort (a 2-week treatment schedule) on tumors. Furthermore, we observed *in vivo* tumors from BAFF-neutralizing antibody-treated ARP-1/MΦ mice, which showed small-sized volumes compared with control IgG2B-treated group with bort as described earlier (Figures 7e and f). These results are correspondent with *in vitro* studies showing that BAFF was involved in MΦ-mediated bort resistance of MM cells.

## Discussion

Mechanisms of bort resistance in MM have been implicated in both intrinsic changes, including MM cells and their subclone heterogeneity, and the protective efficacy of BMSCs.^6, 7, 30^ We demonstrated that primary CD138+ plasma cells from patients with MM underwent spontaneous apoptosis *in vitro*, suggesting that plasma cells *in vivo* reacquired susceptibility when separated from the BM microenvironment *in vitro*. MΦs, a type of BMSCs, were heavily infiltrated in the myeloma microenvironment.^31, 32^ The specific roles of MΦs in the pathogenesis of tumors are now being delineated. For example, a previous study demonstrated that MΦs exhibited tumor-promoting activities via increasing angiogenesis and metastasis, and suppressing anti-tumor immunity.^33, 34^ In particular MΦs could mediate multidrug resistance of MM cells to both conventional and novel chemotherapy drugs.^10^ Our present study demonstrated that PBMC-induced MΦs were resistant to bort *in vitro* and protected primary MM cells and MM cell lines from spontaneous and bort-induced apoptosis. Of note, we found MΦs were not able to reduce panobinostat-induced MM apoptosis. Histone deacetylase inhibitor panobinostat has emerged as a particular treatment option for MM. Previous studies showed the anti-myeloma activity of panobinostat was related to changes in intracellular modifications that influence the interaction of MM cells with the microenvironment.^35^ The positive alteration of panobinostat to MM microenvionment which comprises extracellular matrix and the BMSC may account for the disappeared protective effect of MΦs. We thus assume defining the mechanisms whereby MΦs protected MM cells could potentially identify a promising target for MM therapy.

BAFF, a member of the TNF superfamily, was identified as a humoral factor highly expressed in the BM microenvironment of MM. Studies showed that BMSCs were the main product source of BAFF.^13, 18^Because myeloid lineage cell monocytes including MΦs, dendritic cells were originally found to express BAFF,^36, 37^ and MΦs are important components of the BMSCs of MM that support plasma cell survival and induce chemotherapy resistance.^32^ Therefore, we anticipated a role of BAFF in the adhesion of MΦs and MM cells. Our study demonstrated that MΦs had increased expression of BAFF compared with monocytes, and the secretion level of BAFF in MΦs was higher than that in MM cell lines, which was in accordance with previous reports that BAFF signaling acted mainly through a paracrine system rather than an autocrine mechanism.^13, 17^ We also demonstrated that PBMC-induced MΦs could protect MM cells from both spontaneous and bort-induced apoptosis. When we utilized BAFF-neutralizing antibody under co-culture conditions or knocked-down expression of BAFF on MΦs, MM cell apoptosis significantly increased, implying that BAFF on MΦs could contribute to their ability to confer MM cells with resistance to bort. Therefore, strategies that interfere with BAFF only might be useful to attenuate the resistance of MM cells to bort *in vivo*.

Our data showed that MΦs mediated bort resistance of MM cells, suggesting that the mechanism might be associated with the survival signaling pathway activation of MM cells. Indeed, the present study detected the activation of phosphorylated Akt, Erk1/2 kinase and Src in MM cell lines following co-culture with MΦs, all of which were essential to promote MM cell growth and drug resistance. We also identified that the survival pathway activation was attenuated when the expression of BAFF was interrupted in the co-culture system. Thus, it is plausible that BAFF supports the development of bort resistance of MM cells.

BAFF triggers its functions through NF-*κ*B activation, and two main pathways (canonical and alternative) modulate the activity of NF-*κ*B.^38^ The canonical pathway activation results from the degradation of I*κ*B*α* and thus leads to the nuclear translocation of p65. Activation of the alternative pathway results from IKK*α*-dependent p100 phosphorylation and nuclear translocation of p52. The present study showed that BAFF-induced bort resistance of MM cells/MΦs took place via activation of both classical and alternative NF-*κ*B pathways. This is similar to the interaction between BAFF and its receptors on lymphoma and normal B cells, which promotes I*κ*B*α* degradation and processes of NF-*κ*B2, respectively.^39, 40, 41, 42^

Monoclonal antibody-based therapies existed great promise in MM.^43^Recently, tabalumab (LY2127399), with neutralizing activity against BAFF, was found to be well tolerated and showed a better response when combined with bort in relapsed and refractory patients with MM.^13^ Indeed there are other molecules such as APRIL, BAFF-R,BCMA, and TACI related to BAFF signaling pathway, and particularly APRIL is evidenced playing significant role in MM cell survival and targeting BCMA is in development. Despite these complexities, it remains crucial to examine whether targeting BAFF alone in MM is sufficient, and the overall results highlighted a functional role of BAFF in MΦ-mediated bort resistance of MM cells, providing a basis for the molecular- and immune-targeted therapeutic approach. Taken together, BAFF signaling might serve as an interesting target for MM treatment.

## Materials and methods

### Cell preparation and culture

Human MM cell lines MM.1S, MM.1R, CAG, RPMI8226, ARP-1, and ARK were generously provided by Dr. Qing Yi (Department of Cancer Biology, Lerner Research Institute, Cleveland Clinic, Cleveland, OH, USA). All MM cell lines expressed CD138 (>97% of cells) as detected by the flow cytometric analysis. BM samples were obtained from patients with MM after informed consent from all patients and the approval of the Ethics Committee of the First Affiliated Hospital, College of Medicine, Zhejiang University. Freshly isolated CD138+ cells were purified by positive selection using CD138 microbeads (Miltenyi Biotech, San Diego, CA, USA). All MM cell lines and primary MM cells were maintained in the RPMI-1640 medium containing L-glutamine (Corning Cellgro, Tewksbury, MA, USA), supplemented with 10% fetal bovine serum (FBS; Thermo Fisher Scientific, Gibco, Waltham, MA, USA), at 37 °C and in 5% CO_2_ in air.

PBMCs were obtained from healthy donors after informed consent. Human MΦs were generated from PBMCs *in vitro* as described in a previous study.^31^ Monocytes were cultured at 12–18 million per six-well plates in the RPMI-1640 medium. After 1–2 h of incubation, nonadherent cells were removed and adherent monocytes were cultured in RPMI-1640 containing 10% FBS and M-CSF (10 mg/ml; R&D systems, Minneapolis, MN, USA) for 7 days to transform into MΦs. Before use, MΦs were phenotyped by morphological and detected for classic molecular markers CD68 and CD163.

The MM cells were directly added to MΦs at a 1 : 1 ratio and co-cultured for 24 h with bort to evaluate the effect of MΦs on bort-induced apoptosis in MM cells. Suspended MM cells were obtained by collecting the supernatant and then tested via functional assays.

### Reagents

Primary antibodies against caspase-3, poly(ADP-ribose) polymerase 1 (PARP-1), Akt, phospho-Akt (Ser473), phospho-Akt (Ser308), Src, pSrc (Y416), Erk, pErk (T202/204), and CD68 were purchased from Cell Signaling Technology (Danvers, MA, USA). Anti-CD138 was from Abcam (Cambridge, UK). APC anti-human CD68, PE mouse anti-human CD163, PE mouse anti-human BAFF, APC rat anti-human CD267 (TACI), PE anti-human CD269 (BCMA), and fluorescein isothiocyanate (FITC) mouse anti-human CD268 (BAFF receptor, or BAFF-R) were all obtained from Biolegend (San Diego, CA, USA). Primary antibodies including *β*-actin and glyceraldehyde-3-phosphate dehydrogenase (GAPDH) were obtained from Sigma-Aldrich (Billerica, MA, USA). Horseradish peroxidase (HRP)-conjugated anti-rabbit and anti-mouse antibodies were procured from Jackson ImmunoResearch Laboratories (Lancaster, PA, USA). Human BAFF antibody and mouse immunoglobulin G2b (IgG2B) isotype control were purchased from R&D Systems.

### RNA extraction and quantitative reverse transcriptase-polymerase chain reaction (qRT-PCR) analysis

The total RNA from MM cells was extracted using a Trizol reagent (Invitrogen, Carlsbad, CA, USA). Reverse transcription was performed using a High-Capacity cDNA Reverse Transcription Kit (Applied Biosystems, Waltham, MA, USA). qRT-PCR was performed using the iTaq universal SYBR Green Supermix (Bio-Rad, Hercules, CA, USA) with the Bio-Rad CFX96 real-time system, according to the manufacturer’s instruction, and normalized to GAPDH RNA levels calculated by POWER values and plotted as relative quantification. Each sample was run in triplicate. Amplification primer sequences were as follows: human BAFF, 5′-CGCGGGACTGAAAATCTTTG-3′ and 5′-CACGCTTATTTCTGCTGTTCTGA-3′ human BCMA, 5′-TCCTCTAACATGTCAGCGTTATTGT-3′ and 5′-CATGCCCAGGAGACCTGAT-3′ human TACI, 5′-GGTACCTGCATGTCCTGCAAA-3′ and 5′-TGCAGTCCCTCAGGAGATGGT-3′ human BAFF-R, 5′-TGGGTCTGGTGAGCTGGA-3′ and 5′-CCGGAGACAGAATGATGACCTT-3′ and human GAPDH, 5′-ACGGATTTGGTCGTATTGGGC -3′ and 5′-TTGACGGTGCCATGGAATTG -3′.

### Cell proliferation assay

Cell growth was assessed using a Cell Counting Kit-8 (CCK-8) proliferation assay (Dojindo, Kumamoto, Japan). Cells (5 × 10^3^/100 *μ*l per well) cultured in 96-well plates at indicated times were incubated with CCK-8 for the last 2 h. Then the absorbance was measured at 450 nm using a microplate reader (Bio-Rad, Model 680).

### Enzyme-linked immunosorbent assay

MM cells and MΦs (3 × 10^5^/ml) were cultured alone in six-well plates for 48 h. The supernatants harvested from 48 h cultures and those of BM of 27 patients with MM were measured for soluble BAFF using the Human BAFF/BLyS/TNFSF13B Quantikine enzyme-linked immunosorbent assay (ELISA) Kit (R&D Systems), according to the manufacturer’s instruction. The sensitivity of the kit was 6.44 pg/ml.

### Flow cytometry: cell surface antigens and apoptosis

The expression of BAFF, BCMA, TACI, BAFF-R, CD68, CD163, and CD138 was measured by direct immunofluorescence using APC-conjugated CD68, CD138, TACI; PE-conjugated CD163, BAFF, BCMA; and FITC-conjugated BAFF-R. Each isotype control was determined to exclude the possibility of nonspecific influence. After staining, the cells were washed twice and then suspended in PBS and analyzed using a FACScan flow cytometer (BD Biosciences, San Diego, CA, USA).

The apoptotic cells were measured by staining cells using Annexin V-binding buffer (PharMingen, San Diego, CA, USA), along with Annexin V-FITC/propidium iodide, following the manufacturer’s instructions. After incubating for 10 min at room temperature, the samples were detected by flow cytometry and apoptotic cells were analyzed using FlowJo7.6.1.

### Western blot analysis

The cells were harvested, washed twice with PBS, and extracted using the lysis buffer containing a mixture of protease and phosphatase inhibitor (Thermo Fisher Scientific). The suspension was incubated for 30–60 min at 4 °C, then centrifuged at 16 000 r.p.m. for 30 min at 4 °C. The supernatant was then used as whole-cell lysates. The protein concentration was determined using the Bio-Rad Protein Assay. The samples were boiled at 95 °C for 5 min after mixing with a 4 × sodium dodecyl sulfate (SDS) loading buffer (Invitrogen). The proteins (20–40 *μ*g) were subjected to 10% SDS-polyacrylamide gel electrophoresis and subsequently transferred to a polyvinylidene difluoride membrane (Merck Millipore, Darmstadt, Germany). The membranes were blocked with 5% bovine serum albumin for 1–2 h at room temperature. Then, the blots were incubated with primary antibodies overnight at 4 °C. Immunoblots were washed with Tris-buffered saline with Tween (TBST) buffer three times and incubated with HRP-conjugated anti-mouse or anti-rabbit antibodies (1 : 5000) for 1 h at room temperature, followed by TBST washing three times and subsequent autoradiography with the ChemiDoc MP Imaging System (Bio-Rad) using an enhanced chemiluminescence detection kit (Biological Industries Israel Beit Haemek Ltd., Kibbutz Beit Hamek, Israel).

### RNA interference

PBMC-induced MΦs were transiently transfected with three siRNAs (TNSF13B-Homo-296:5′-CGCCUUACUUCUUGCCUUATT-3′ TNSF13B-Homo-700:5′-CUGCUUGCAACUGAUUGCATT-3′ TNSF13B-Homo-973:5′-GCCUGAAACACUACCCAAUTT-3′) against human BAFF (TNAF13B) and a scrambled nontargeting siRNA (GenePharm, Shanghai, China) using Lipo2000 (Life Technology, Pittsburgh, PA, USA). The BAFF-specific·siRNAt-ransfected MΦs were co-cultured with MM cells with or without bort, and then evaluated in functional studies.

### Immunofluorescence and immunohistochemistry analyses

Paraformaldehyde-fixed, Triton X-100 permeabilized cells of the BM biopsy tissues from patients with MM were used for immunofluorescence staining to analyze the expression of BAFF in CD68-expressing MΦs. Also, paraformaldehyde-fixed, paraffin-embedded sections (5 *μ*m) of tumor tissues from tumor-bearing NOD-SCID (nonobese diabetic-severe combined immunodeficient) mice were used for immunohistochemistry staining to analyze CD68-expressing MΦs and cleaved PARP (apoptotic tumor cells) as described earlier.^44^

### MΦ-mediated bort resistance of MM cells *in vivo*

Four-week-old female NOD-SCID mice were obtained from Vital River Laboratory Animal Technology Co. Ltd. (Beijing, China) and housed in the animal facility of Zhejiang University School of Medicine. The Tab of Animal Experimental Ethical Inspection of the First Affiliated Hospital, College of Medicine, Zhejiang University approved the procedures and protocols of all experiments. The mice were subcutaneously injected in the right flank with one million ARP-1 (control group) and one million ARP-1/two million monocytes both suspended in 100 *μ*l of PBS. After palpable tumors (tumor diameter ⩾5 mm) developed, they were harvested for immunohistochemistry staining of CD68 to determine the infiltration of MΦs. Some mice received intraperitoneal injections of bort (2 μg/mouse, every 3 days) for 2 weeks, and injections of PBS served as a control. In some experiments, the mice received intraperitoneal injections of BAFF-neutralizing antibody or control IgG2B (100 mg/mouse, every 3 days), and administered with bort as described earlier. Tumor sizes were measured every 3 days using calipers and calculated using the formula *V*=1/2 (length × width^2^).

### Statistical analysis

Data were analyzed using the GraphPad Prism 6 (GraphPad Software, LaJolla, CA, USA) and Microsoft Office Excel. All results were expressed as mean±standard deviation (S.D.), and the statistical differences among two groups were determined using a two-tailed Student’s *t*-test. All *P*-values <0.05 were recognized as statistically significant. All experiments were performed in triplicate and three or more independent assays. *P<0.05, **P<0.01, ***P<0.001.

## Publisher’s Note

Springer Nature remains neutral with regard to jurisdictional claims in published maps and institutional affiliations.
