# Supplementary figures and images for: BAFF is involved in macrophage-induced bortezomib resistance in myeloma
Source: Cell Death Dis. 2017 Nov 2;8(11):e3161–. doi: 10.1038/cddis.2017.533 (PMC5775406; doi:10.1038/cddis.2017.533)

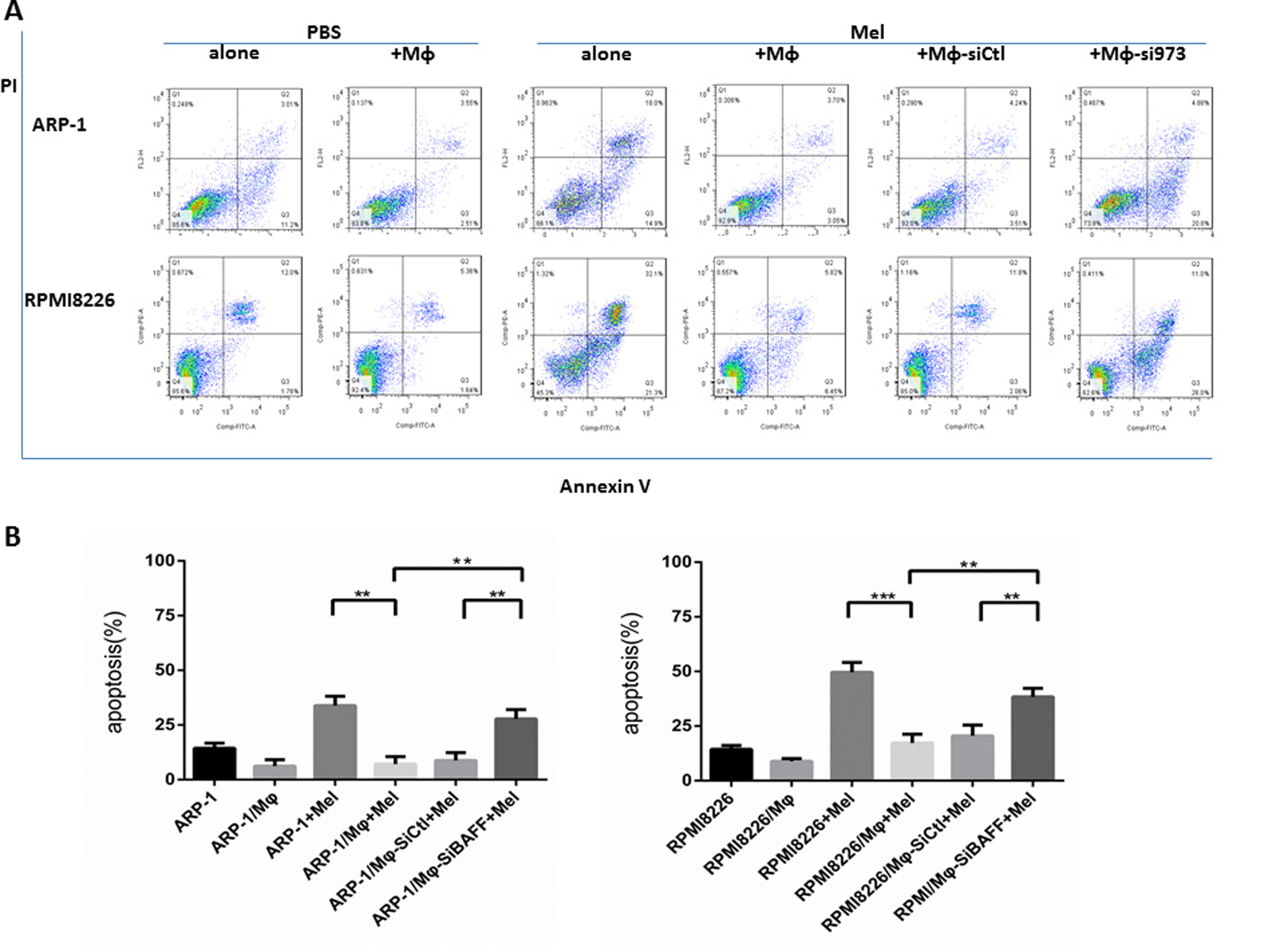

Supplement: Supplementary Figure 1 [file cddis2017533x1.tif]

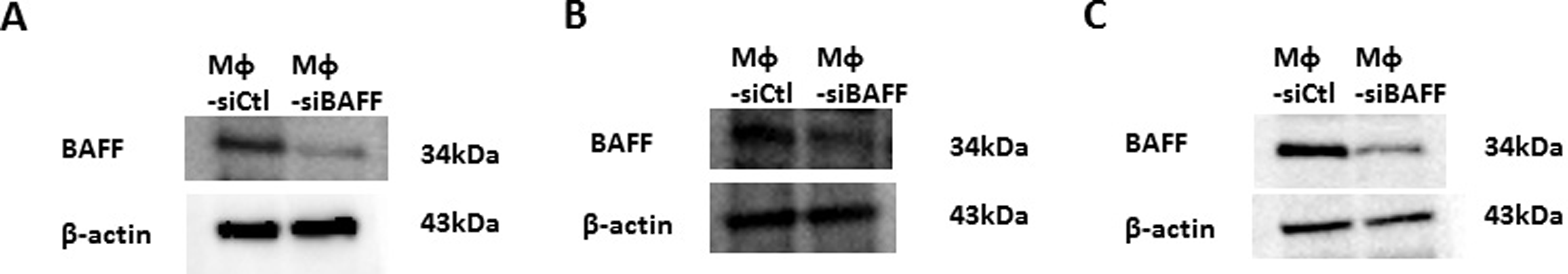

Supplement: Supplementary Figure 2 [file cddis2017533x2.tif]
