# Supplementary material for: BAFF is involved in macrophage-induced bortezomib resistance in myeloma
Source: Cell Death Dis. 2017 Nov 2;8(11):e3161–. doi: 10.1038/cddis.2017.533 (PMC5775406; doi:10.1038/cddis.2017.533)
Supplement: Supplementary Figure Legends [file cddis2017533x3.docx]

**Supplementary Figure 1**

(A) A representative result showing melphalan-induced apoptosis on ARP-1 cells cocultured with MΦs following the BAFF-specific·siRNAs using flow cytometry analysis. The BAFF knockdown effect resulted in reduced ability of MΦs in protecting MM cells. (B) Result showing percentage of melphalan-induced apoptotic MM cells (ARP-1 and RPMI8226) in direct co-culture with BAFF-knocked down MΦs. Values are presented as means ±SD.**P* < 0.05, ***P* < 0.01.

**Supplementary Figure 2**

(A, B, C) MΦs treated with BAFF-specific siRNAs showed a diverse reduction of BAFF protein compared with nontargeting siRNA (control) at 72 h using Western blot, with β-actin as a loading control.
